# Effects of Exogenous (K^+^) Potassium Application on Plant Hormones in the Roots of *Tamarix ramosissima* under NaCl Stress

**DOI:** 10.3390/genes13101803

**Published:** 2022-10-06

**Authors:** Yahui Chen, Shiyang Zhang, Shanfeng Du, Guangyu Wang, Jinchi Zhang, Jiang Jiang

**Affiliations:** 1 Collaborative Innovation Center of Sustainable Forestry in Southern China of Jiangsu Province, Nanjing Forestry University, Nanjing 210037, China; 2 Department of Forest Resources Management, Faculty of Science, University of British Columbia, Vancouver, BC V6T 1Z4, Canada

**Keywords:** *Tamarix ramosissima*, NaCl stress, exogenous potassium, plant hormone, NaCl poison

## Abstract

Abiotic stresses such as salt stress seriously affect the growth and yield of plants. *Tamarix ramosissima* Lcdcb (*T. ramosissima*) is a widely cultivated halophyte in saline-alkali areas of the world. As an essential element for plant growth and development, K^+^ plays an irreplaceable role in improving the tolerance of plants to salt stress. However, there are few reports on the mechanism of K^+^ in promoting plant hormones to reduce the damage of NaCl stress to *T. ramosissima*. In this study, we sequenced the transcriptome of the roots of *T. ramosissima* which were treated with exogenous potassium (K^+^) for 0 h, 48 h and 168 h under NaCl stress, according to the changes in the expression levels of differentially expressed genes (DEGs) in *T. ramosissima* roots. Key candidate genes and metabolic pathways related to plant hormones were mined for analysis and further verified by quantitative real-time PCR (qRT-PCR). The results showed that under NaCl stress for 48 h and 168 h, there were a large number of DEGs in the roots of *T. ramosissima*, and the expression levels changed over time. In particular, we found that 56 plant hormone-related genes were annotated to the Kyoto Encyclopedia of Genes and Genomes (KEGG) pathway, and with the increase of time, their expression levels were mainly up-regulated and involved in the related metabolic pathways to resist NaCl stress. It is worth noting that 7 DEGs related to abscisic acid (ABA), 28 DEGs related to auxin, 1 DEG related to ethylene (ET), and 1 DEG related to cytokinin (CK) were added within 168 h of exogenous potassium, and they were involved in alleviating the root damage of *T. ramosissima* under NaCl stress and played an important role. In addition, we found the plant hormone signal transduction pathway, which plays an important role in resistance to NaCl stress. As a result of this study, the molecular mechanism of plant hormones involved in applying exogenous potassium under NaCl stress is further understood, resulting in a better understanding of how exogenous potassium can alleviate the damage caused by NaCl under stress in *T. ramosissima*.

## 1. Introduction

Plant growth, development and yield are negatively affected by abiotic stresses such as drought, salinity, low and high temperature [1]. In particular, salt stress is the main factor restricting plant growth [2]. Research shows that excessive NaCl accumulation in soil and water is the main salt damage that inhibits plant growth [3,4]. Plant physiological processes are affected under salt stress [5], for example, photosynthesis, protein synthesis, energy and lipid metabolism, etc. In general, the destructive effects of salt stress on plants are mainly achieved through processes such as osmotic stress, ion toxicity, nutrient imbalance and oxidative stress [6]. Eventually, plant growth is inhibited, and in severe cases, plant death [7].

Plants produce a class of important substances known as phytohormones. At low concentrations, they can have an effect on physiological processes such as plant growth and development, metabolism, and environmental response. [8]. Plant hormones are key regulators in various physiological processes and one of the most important small signaling molecules affecting plant growth and yield [9]. Plant hormones trigger adaptive responses caused by external stimuli, such as biotic and abiotic stresses [10,11,12]. Interestingly, regulating plant hormones can effectively alleviate salt stress during salt stress [13]. Therefore, using plant hormones is an effective measure to improve plant growth and alleviate salt stress [14], and they can regulate plant growth and development at homeostasis [15]. According to the function of plant hormones, it can be divided into two categories: the first category of plant hormones includes auxin, gibberellin, cytokinin (CK), brassinolide, jasmonic acid (JA) and strigolactone. This class of plant hormones can play important roles in growth-promoting activities through cell division, cell differentiation, elongation, patterning, stomatal movement, flowering, and seed germination and development. The second class of plant hormones includes abscisic acid (ABA), salicylic acid (SA), and jasmonic acid. They are mainly involved in biotic and abiotic stress responses under different environmental conditions such as sunlight, soil conditions, soil moisture and nutrients [16,17,18]. Studies have reported that plants can synthesize ABA in large quantities after being subjected to salt stress, it can reduce plant stomatal conductance, water consumption, wilting and transpiration, thereby maintaining low Na^+^/K^+^ and enhancing the activity of protective enzymes, ultimately maintaining the integrity of cell membrane structure and reducing salt-induced damage [19]. Additionally, the application of exogenous abscisic acid can improve the tolerance of plants to salt [20], and it can also improve the ability of plants to grow under salt stress [21]. In salt medium, after exogenous IAA was applied to *Solanum tuberosum*, the in vitro cultured plants could better regulate osmotic pressure, maintain plant nutrient homeostasis, and operate the antioxidant system, thereby improving plant growth [22]. In addition to enhancing mineral homeostasis (K^+^, Ca^2+^, and Mg^2+^) and Na^+^ transport, exogenous IAA can also inhibit excessive Na^+^ accumulation in roots under NaCl stress. [23]. Aside from regulating plant growth and defense under NaCl stress, jasmonic acid can also interact with growth-related hormones [24]. For example, applying exogenous MeJA can alleviate the adverse effects of NaCl on soybean growth and endogenous hormones, and enhance soybean yield’s salt tolerance of soybean [25]. Application of exogenous JA can enhance the activity of antioxidant enzymes to inhibit the excess reactive oxygen species caused by salt stress, thus effectively protecting wheat seedlings from the damage of NaCl stress [26]. Interestingly, cytokinins (CKs) play important and complex roles in plant growth and abiotic stress responses [27]. The application of exogenous CKs can not only improve the salt tolerance of wheat seedlings, but also increase the sensitivity of potatoes to salt [28,29]. CKs in plants can also enhance resistance to salt by regulating the division and expansion of endosperm cells [30]. CKs can play a role in alleviating salt-induced senescence and maintaining crop yield under salt stress [31]. Consequently, applying exogenous CKs to plants can increase plants’ salt tolerance of plants [32]. Notably, ethylene is largely able to improve Na^+^/K^+^ homeostasis to promote salt tolerance [33], and ethylene signaling can also promote salt tolerance in *Arabidopsis* [34].

K^+^ is the most abundant cation in plants and is essential for plant nutrition, growth, enzyme homeostasis and osmotic pressure regulation [35]. Under salt stress, Na^+^ competes with K^+^ for absorption through the cytoplasmic membrane of the root, which enables ROS to activate the K^+^ permeation channel in the root to cause K^+^ efflux, reduce the K^+^/Na^+^ ratio, and ultimately lead to the toxicity of Na^+^ to plants [36]. Under NaCl stress, the absorption of K^+^ can better maintain the K^+^/Na^+^ ratio and improve the salt tolerance of plants [37]. For example, overexpression of *OsHAK1* in rice increased K^+^ uptake and K^+^/Na^+^ ratio, indicating that *OsHAK1* plays an important role in K^+^-mediated rice growth and salt tolerance at low and high K^+^ concentrations [38]. Increasing the K^+^ accumulation level of transgenic plants under NaCl stress and ROS scavenging ability of transgenic plants was associated with improved tolerance to NaCl stress following *ApKUP4* overexpression in *Arabidopsis thaliana* [39]. Exogenous potassium was applied to *T. ramosissima* roots under NaCl stress for 48 h and 168 h. In addition to helping *T. ramosissima* overcome oxidative damage, K^+^ improved root K^+^ absorption and alleviated NaCl toxicity [40].

Halophytes are highly salt-tolerant plants that can complete their life cycle at ≥200 mM NaCl concentration [41]. *Tamarix* plants are halophilic plants [42], they are able to avoid salt damage by secreting salt through the salt glands [43,44], and the use of physiological and metabolic processes to enhance tolerance [45,46,47], such as osmoregulation, free radical scavenging, cellular detoxification and protection of biological macromolecules [48]. *T. ramosissima*, a member of the genus *Tamarix* plants, is widely grown in saline soils [49]. Studies have reported that high salt stress will limit plant growth even more [50]. *T. ramosissima* promotes its growth under ≤100 mM NaCl stress, but inhibits its growth under ≥200 Mm NaCl stress [51]. However, as a halophyte, *T. ramosissima* has the ability to retain more K^+^ under salt stress conditions to adapt to different saline-alkali conditions [52]. It has been reported that the application of 10 Mm KCl under the stress of 200 mM NaCl can increase the activities of SOD, POD and CAT in leaves of *T. ramosissima* [53], and K^+^ transporter, K^+^ channel and amino acid-related genes in *T. ramosissima* root are up-regulated and related genes are involved in resisting NaCl stress and maintaining the growth of *T. ramosissima* [40,54]. In this study, *T. ramosissima* was taken as the research object, and the important metabolic pathways and key differentially expressed genes (DEGs) related to plant hormones in response to NaCl stress were excavated from the transcriptome level by exogenous potassium application. The purpose of this study is to provide a scientific theoretical foundation for future research on the breeding of salt-tolerant *Tamarix* species under NaCl stress, as well as the effect of K+ on alleviating NaCl toxicity by using exogenous potassium.

## 2. Materials and Methods

### 2.1. Plant Materials

The 5-month-old *T. ramosissima* seedlings with similar growth were transferred to a 24-well hydroponic box (40 cm × 30 cm × 16 cm) filled with 1/2 Hoagland nutrient solution and placed at a temperature of 26 ± 2 °C, with relative humidity of 40% to 55%. The culture medium was replaced every 3 days, and the culture medium was used for 2 months after cultivation. The experiment was carried out in the key laboratory of the School of Forestry, Nanjing Forestry University.

### 2.2. Treatment of Experimental Seedlings

In this study, 1/2 Hoagland nutrient solution was used as the control group, 1/2 Hoagland nutrient solution supplemented with 200 mM NaCl and 1/2 Hoagland nutrient solution supplemented with 200 mM NaCl + 10 mM KCl was used as the treatment group. The nutrient solution for each treatment group was replaced every 3 days. Culture medium, 8 plants in each group, a total of 3 replicates, and 24 seedlings. In this study, we collected root samples at 0 h, 48 h, and 168 h of treatment, and placed them in liquid nitrogen immediately after treatment, and then transferred them to a −80 °C refrigerator for future use.

### 2.3. Transcriptome Sequencing and Differentially Expressed Gene Screening

We sent the test samples processed in 2.2 to Guangzhou GENE Denovo Company for high-throughput transcriptome sequencing. Firstly, we used the Omega kit (Beinuo Bio, Shanghai, China) from Omega Bio-Tek to extract the total RNA. Then, we did an RNA quality assessment on an Agilent 2100 Bioanalyzer (Agilent Technologies, Palo Alto, CA, USA). We also used RNase-free agarose gel electrophoresis to check the quality as well. After the RNA extraction process, eukaryotic mRNA was enriched by Oligo(dT) beads, and prokaryotic mRNA was enriched by removing rRNA by Ribo-ZeroTM Magnetic Kit (Epicentre, Madison, WI, USA). After that, by using the fragmentation buffer, the enriched RNA was divided into short fragments, and we reversed transcripted it into cDNA with random primers. Second-strand cDNA was synthesized by DNA polymerase I, Rnase H, dNTP and a buffer. The cDNA fragments were purified with a QiaQuick PCR extraction kit (Qiagen, Venlo, The Netherlands), end-repaired, poly(A) was added and ligated to Illumina sequencing adapters. They were sequenced using Illumina HiSeq^TM^4000 by Gene Denovo Biotechnology Company (Guangzhou, China). We submitted the raw data obtained by transcriptome sequencing to the National Center for Biotechnology Information (NCBI) Short Reads Archive (SRA) database (SRP Number: SRP356215). Transcriptome data were analyzed and differentially expressed genes were screened according to the method of Chen et al. [40,54].

### 2.4. Quantitative Real-Time PCR (qRT-PCR) Validation

For the purpose of verifying the accuracy of RNA-Seq results, we randomly selected 7 candidate genes. To detect key candidate genes, qRT-PCR primers were designed (Supplementary Table S1). ABI ViiATM 7 Real-time PCR system (ABI, Carlsbad, CA, USA) was used for the qRT-PCR detection of target genes according to the method of Chen et al. [49]. Three times each gene was replicated technically and three times each gene was replicated biologically. The internal reference gene tubulin was used, and the relative expression was calculated by the 2^−ΔΔCt^ method [53].

### 2.5. Experiment Data Processing

In this study, Data statistics were performed using Excel (Microsoft, Washington, DC, USA), phylogenetic trees were created using MEGA 11 software (MEGA Software, Erie, PA, USA) and graphs were drawn using Origin 2018 software (OriginLab Corporation, Northampton, MA, USA). For transcriptome sequencing detection, 3 technological and 3 biological repetitions were included.

## 3. Results

### 3.1. Screening of Differentially Expressed Genes (DEGs)

The data obtained by transcriptome sequencing were screened according to FDR < 0.05 and Log_2_ fold-change > 1 as up-regulated DEGs, and FDR < 0.05 and Log_2_ fold-change < −1 as down-regulated DEGs. In this study (Supplementary Figure S1), except for the control group-0 h vs. 200 mM NaCl-48 h and the control group-0 h vs. 200 mM NaCl-168 h comparison group, the number of DEGs was up-regulated more than down-regulated, the DEGs for the remaining comparison groups were all have more down-regulated than those which were up-regulated. Among them, 13,833 DEGs were identified in the 200 mM NaCl-48 h vs. 200 mM NaCl + 10 mM KCl-48 h comparison group, including 5491 DEGs that were up-regulated and 8342 DEGs that were down-regulated. In the 200 mM NaCl-168 h vs. 200 mM NaCl + 10 mM KCl-168 h comparison group, 10,818 DEGs were identified, including 4392 DEGs that were up-regulated and 6426 DEGs that were down-regulated.

### 3.2. Kyoto Encyclopedia of Genes and Genomes (KEGG) Pathway Analysis

**Figure 1:**
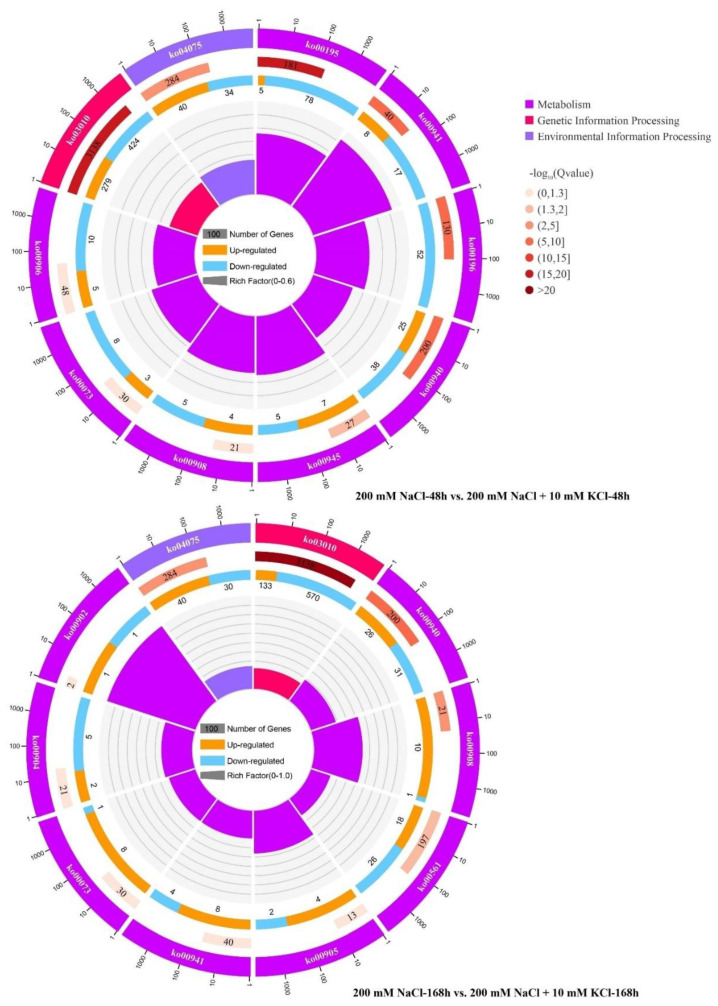
Top 10 KEGG pathway analysis. (KEGG pathway analysis of the top 10 enrichment of the root of *T. ramosissima* under NaCl stress for 48 h and 168 h with exogenous potassium. The first and outer circles show the top 20 KEGG pathways enriched, while the scale outside the circle indicates the number of genes. Different colours represent different ontologies. Next, the KEGG pathway number in the background gene along with the Q value can be seen in the second circle. A darker colour indicates a lower Q value, and a lighter colour indicates a higher Q value. Longer bars indicate more genes. Dark colours indicate genes that are upregulated, and light colours indicate genes that are downregulated. Below is a display of the specific value. Last and inner circle: rich factor values for KEGG pathways (the number of differential genes in this pathway divided by all numbers); background grid lines (each grid represents 0.1)).

When *T. ramosissima* roots were exposed to exogenous potassium for 48 h and 168 h under NaCl stress, the 10 most significant Kyoto Encyclopedia of Genes and Genomes (KEGG) pathways (*p* < 0.05) were significantly enriched (Table 1). Six co-annotated metabolic pathways were found in the comparison groups of 200 mM NaCl-48 h vs. 200 mM NaCl + 10 mM KCl-48 h and 200 mM NaCl-168 h vs. 200 mM NaCl + 10 mM KCl-168 h, which were ribosome pathway, flavonoid biosynthesis pathway, phenylpropanoid biosynthesis pathway, plant hormone signal transduction pathway, zeatin biosynthesis pathway and cutin, suberine and wax biosynthesis pathway. In the comparison group of 200 mM NaCl-48 h vs. 200 mM NaCl + 10 mM KCl-48 h, the number of up-regulated DEGs of the two metabolic pathways including plant hormone signal transduction pathway and stilbenoid, diarylheptanoid and gingerol biosynthesis pathway was greater than the number of down-regulated DEGs, the remaining 8 metabolic pathways are that the number of down-regulated DEGs is greater than the number of up-regulated DEGs (Figure 1). In 200 mM NaCl-168 h vs. 200 mM NaCl + 10 mM KCl-168 h comparison group, four metabolic pathways (plant hormone signal transduction pathway, brassinosteroid biosynthesis pathway, flavonoid biosynthesis pathway and cutin, suberine and wax biosynthesis pathway) have more up-regulated DEGs than down-regulated DEGs. The remaining six metabolic pathways are that the number of down-regulated DEGs is greater than the number of up-regulated DEGs (Figure 1). It is worth noting that the number of up-regulated DEGs was always greater than the number of down-regulated DEGs in the Plant hormone signal transduction pathway when exogenous potassium was applied for 48 h and 168 h under NaCl stress. In conclusion, the Plant hormone signal transduction pathway has a large number of up-regulated and DEGs that have been involved in resisting the damage of NaCl stress to *T. ramosissima*, and the Plant hormone signal transduction pathway plays an important role in alleviating the injury caused by NaCl stress and maintaining the normal growth of *T. ramosissima*.

### 3.3. Plant Hormone Signal Transduction Pathway Analysis

**Figure 2:**
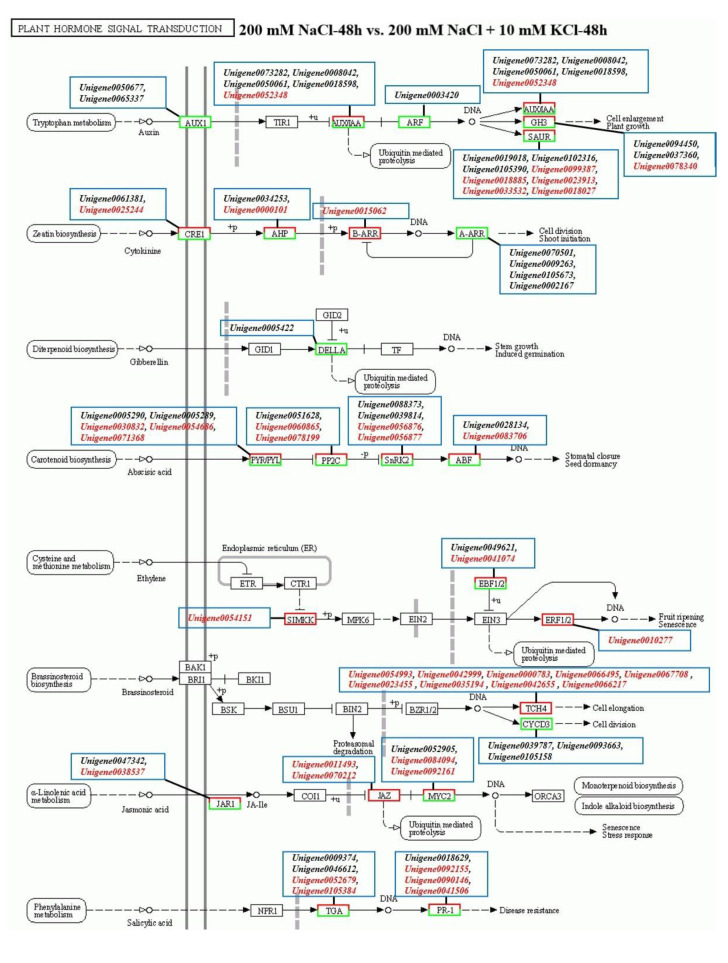

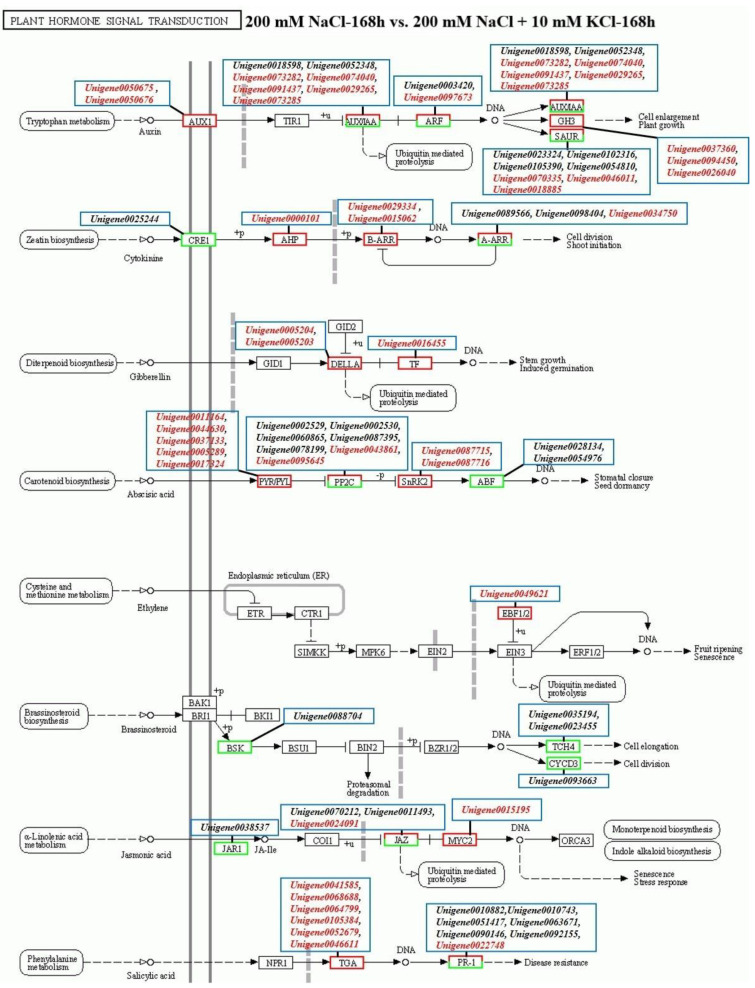
Plant hormone signal transduction pathway. (Exogenous potassium was applied to the roots of *T. ramosissima* for 48 h and 168 h under NaCl stress, and the gene expression changes were annotated to signal transduction pathways associated with plant hormones. The black pixels indicate DEGs whose expression levels are down-regulated, and the red pixels indicate DEGs whose expression levels are up-regulated).

**Figure 3:**
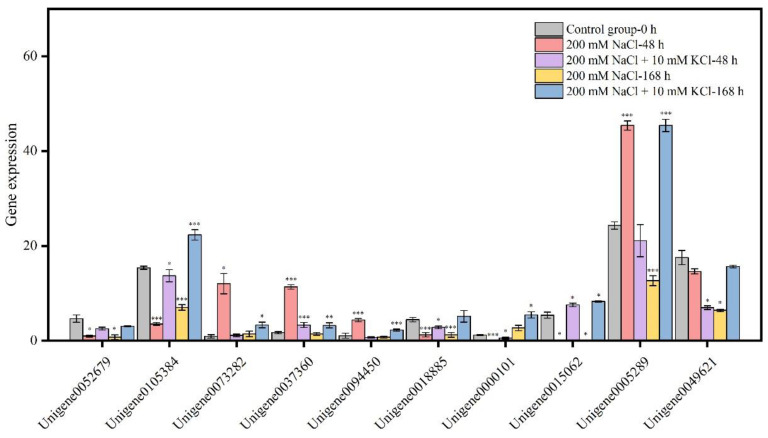
Changes in the expression of key DEGs in the plant hormone signal transduction pathway. (The roots of *T. ramosissima* were exposed to exogenous potassium for 48 h and 168 h under NaCl stress, and the expression levels of 10 key candidate genes in the Plant hormone signal transduction pathway changed. Note: *p* ≥ 0.05 is not marked; 0.01 < *p* < 0.05 is marked as *; 0.001 < *p* < 0.01 is marked as **; *p* ≤ 0.001 is marked as ***).

According to the analysis of the Plant hormone signal transduction pathway (Figure 2), the results showed that 74 DEGs were annotated in the 200 mM NaCl-48 h vs. 200 mM NaCl + 10 mM KCl-48 h comparison group. Among them, 40 DEGs were up-regulated and 34 DEGs were down-regulated. In the 200 mM NaCl-168 h vs. 200 mM NaCl + 10 mM KCl-168 h comparison group, 70 DEGs were annotated. Among them, 40 DEGs were up-regulated and 30 DEGs were down-regulated. Specially, there were 27 DEGs co-annotated to the 200 mM NaCl-48 h vs. 200 mM NaCl + 10 mM KCl-48 h and 200 mM NaCl-168 h vs. 200 mM NaCl + 10 mM KCl-168 h comparison groups (Supplementary Figure S2). Among them, the expression levels of *Unigene0073282*, *Unigene0037360*, *Unigene0094450*, *Unigene0005289* and *Unigene0049621* were down-regulated when exogenous potassium was applied for 48 h under NaCl stress, but the expression levels of the comparison group were up-regulated when exogenous potassium was applied under NaCl stress for 168 h. It is remarkable that the expression levels of *Unigene0052679*, *Unigene0105384*, *Unigene0018885*, *Unigene0000101* and *Unigene0015062* were all up-regulated at 48 h and 168 h under NaCl stress (Figure 3). Therefore, these 10 DEGs (Supplementary Table S2) are affected by exogenous addition, actively participate in resistance to NaCl stress, alleviate NaCl injury, and play an important role in the plant hormone signal transduction pathway.

### 3.4. Analysis of Candidate Genes Related to Plant Hormones in the Roots of T. ramosissima by Exogenous Potassium Application under NaCl Stress

**Figure 4:**
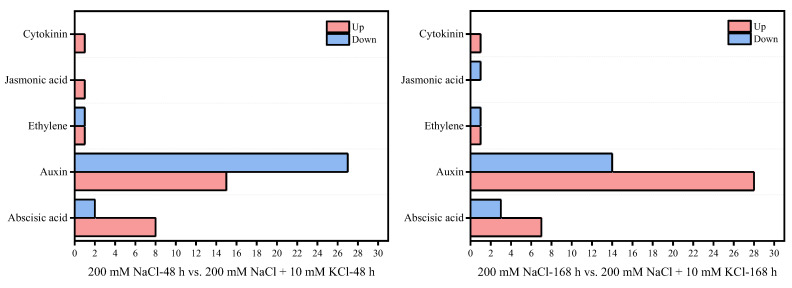
Statistical chart of the expression number of key plant hormone genes in the roots of *T. ramosissima* by exogenous potassium application under NaCl stress. (The number of up-regulated and down-regulated changes of 56 plant hormone key genes found in the roots of *T. ramosissima* under NaCl stress for 48 h and 168 h with exogenous potassium).

The roots of *T. ramosissima* were exposed to exogenous potassium for 48 h and 168 h under NaCl stress, and 56 Plant hormone-related genes were found to be annotated to the KEGG pathway (Table 2), including 10 genes for abscisic acid (ABA), auxin 42 genes, 2 genes for ethylene (ET), 1 gene for jasmonic acid (JA) and 1 gene for cytokinin (CK). The expression levels of these genes changed over time. In the 200 mM NaCl-48 h vs. 200 mM NaCl + 10 mM KCl-48 h comparison group, 26 DEGs were up-regulated and 30 DEGs were down-regulated. Among them, in the comparative analysis of 200 mM NaCl-48 h vs. 200 mM NaCl + 10 mM KCl-48 h, the number of up-regulated DEGs were ABA (8), auxin (15), ET (1), JA (1) and CK (1), the number of down-regulated DEGs were ABA (2), auxin (27) and ET (1) (Figure 4). In the comparative analysis of 200 mM NaCl-168 h vs. 200 mM NaCl + 10 mM KCl-168 h, 37 DEGs were up-regulated and 19 differentially expressed genes were down-regulated. Among them, in the comparative analysis of 200 mM NaCl-168 h vs. 200 mM NaCl + 10 mM KCl-168 h, the number of up-regulated DEGs were ABA (7), auxin (28), ET (1) and CK (1), the number of down-regulated DEGs was ABA (3), auxin (14), ET (1) and JA (1) (Figure 4).

According to the Log_2_ fold-change analysis of 56 plant hormone -related genes (Table 2), when exogenous potassium was applied for 48 h under NaCl stress, the expression levels of these 19 DEGs were down-regulated, including 1 DEG in abscisic acid (*Unigene0039063*) and 18 DEGs in auxin (*Unigene0000292*, *Unigene0008042*, *Unigene0009423*, *Unigene0029265*, *Unigene0046011*, *Unigene0047953*, *Unigene0048320*, *Unigene0048945*, *Unigene0050675*, *Unigene0050677*, *Unigene0059004*, *Unigene0059322*, *Unigene0065339*, *Unigene0073282*, *Unigene0074040*, *Unigene0089917*, *Unigene0091437* and *Unigene0097673*). Nevertheless, these 19 DEGs were up-regulated when exogenous potassium was applied for 168 h under NaCl stress. Significantly, Under NaCl stress, the expression levels of exogenous potassium were up-regulated at 48 h and 168 h, including 6 DEGs in ABA (*Unigene0079211*, *Unigene0063711*, *Unigene0008844, Unigene0080236*, *Unigene0044630* and *Unigene0071368*) and 10 DEGs in auxin (*Unigene0018027*, Unigene0018885, *Unigene0038393*, *Unigene0050676*, *Unigene0059399*, *Unigene0070335*, *Unigene0071678*, *Unigene0072291*, *Unigene0073285*, *Unigene00775575*), 1 DEG in ET (*Unigene0010277*) and CK (*Unigene0045738*). The results showed that these 18 plant hormone candidate genes played an important role at 48 h and 168 h of exogenous potassium alleviating NaCl injury, and the plant hormone-related genes increased with time, and the up-regulated expression of differentially expressed genes increased under NaCl stress for 48 h and 168 h under NaCl stress. In brief, during the 168 h application of exogenous potassium under NaCl stress, the expression levels of plant hormone-related genes were mainly up-regulated, and the corresponding mechanisms were regulated to resist NaCl stress.

### 3.5. Phylogenetic Tree Analysis of Auxin Key Candidate Genes in Plant Hormones

According to the data analysis in 3.4, this study found that a large number of auxin-related genes are involved in the resistance of plant hormones to NaCl stress. In particular, we found 28 genes (*Unigene0000292*, *Unigene0008042*, *Unigene0009423*, *Unigene0029265*, *Unigene0046011*, *Unigene0047953*, *Unigene0048320*, *Unigene0048945*, *Unigene0050675*, *Unigene0050677*, *Unigene0059004*, *Unigene0059322*, *Unigene0065339*, *Unigene0073282*, *Unigene0074040*, *Unigene0089917*, *Unigene0091437*, *Unigene0097673*, *Unigene0018027*, *Unigene0018885*, *Unigene0038393*, *Unigene0050676*, *Unigene0059399*, *Unigene0070335*, *Unigene0071678*, *Unigene0072291*, *Unigene0073285* and *Unigene0077555*) whose expression levels were up-regulated under NaCl stress with application of exogenous potassium for 168 h. Therefore, we selected these 28 genes as key candidate genes for auxin and compared their protein and amino acid sequences on National Center for Biotechnology Information (NCBI) using BLAST, and selected 25 homologous gene species (Table 3). Using MEGA software (MEGA Software, Pennsylvania, USA), the 28 amino acid protein sequences of the key candidate genes in *T*. *ramosissima*’s auxin and those of the 25 homologous protein genes were combined to construct a phylogenetic tree for analysis. The results showed that *Unigene0073285* was closely related to *Sesamum indicum*, *Unigene0050677* was closely related to *Quercus suber*, *Unigene0050675* was closely related to *Pistacia vera*, *Unigene0089917* was closely related to *Malus domestica*, *Unigene0046011* was closely related to *Cucumis sativus*, *Unigene0059399* was closely related to *Momordica charantia*, *Unigene0059004* was closely related to *Mangifera indica*, *Unigene0048320* was closely related to *Rosa chinensis*, *Unigene0070335* was closely related to *Camellia sinensis*, and *Unigene0048945* was closely related to *Pyrus bretschneideri* (Supplementary Figure S3).

### 3.6. Quantitative Real-Time PCR (qRT-PCR) Validation of DEGs

**Figure 5:**
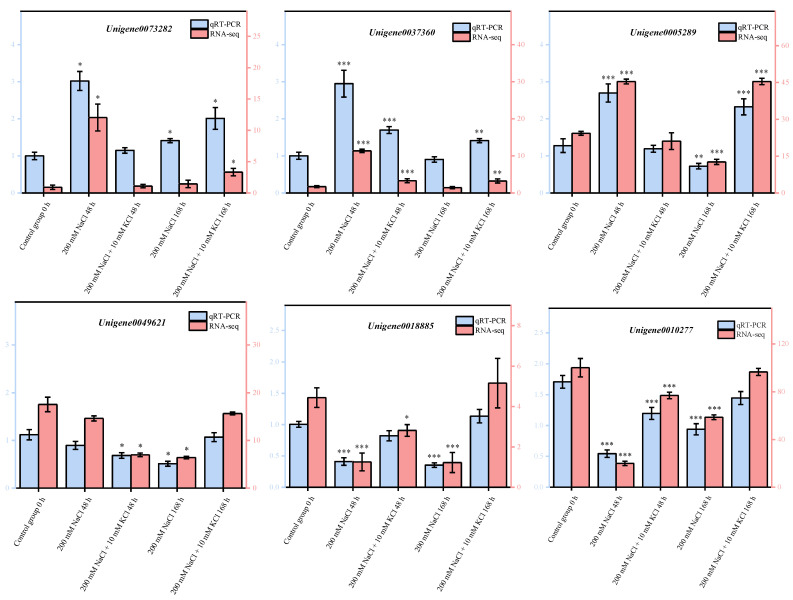

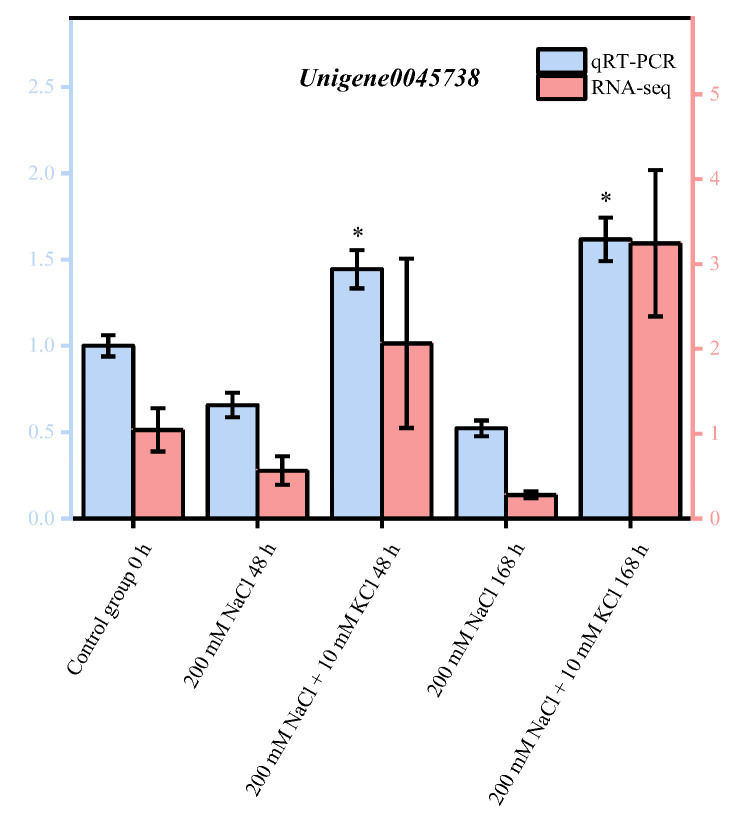
Validation of DEGs by qRT-PCR. (7 DEGs were randomly selected for qRT-PCR validation, and the error bars were obtained from multiple replicates of qRT-PCR. Note: *p* ≥ 0.05 is not marked; 0.01 < *p* < 0.05 is marked as *; 0.001 < *p* < 0.01 is marked as **; *p* ≤ 0.001 is marked as ***; Blue color Numerical value has been shown on the left side of the *Y* axis; Red color: Numerical value has been shown on the right side of the *Y* axis).

To verify the reliability of transcriptome sequencing data, we randomly selected 7 key DEGs for qRT-PCR verification using Chen et al. [54]. According to these validation results, qRT-PCR results were completely consistent with transcriptome sequencing analysis results (Figure 5), proving that the transcriptome data are accurate and reliable. The research could provide a scientific foundation that exogenous potassium can be applied to *T. ramosissima* roots to relieve NaCl stress injury by excavating key candidate genes of plant hormones and salt tolerance genes.

## 4. Discussion

Plants are often subjected to environmental stresses such as high temperature and salt stress, which adversely affect the seed germination, growth and development of plants [55]. However, various signal transduction pathways of plant hormones in response to abiotic stress play a key role in regulating plant growth and development [18]. In this study, the plant hormones which we focused on were abscisic acid (ABA), auxin, Ethylene (ET), jasmonic acid (JA), and cytokinin (CK). Due to the complex interactions between different plant hormones, they can regulate many physiological processes. We studied the expression of key plant hormone genes in the roots of *T. ramosissima* in response to the application of exogenous potassium under NaCl stress.

Plant hormones, as the main signals in plants, are involved in the response of plants to salt stress [56]. Among them, auxin, ABA, JA, and CK regulate plant responses to salt stress and counteract the adverse effects of stress conditions [57]. Under salt stress, the content of ABA in plants increases, and the salt tolerance of plants is enhanced by stimulating stomatal closure, regulating gene expression, and increasing osmotic compatible substances [58]. In particular, under NaCl stress, applying exogenous abscisic acid in rice reduced the Na^+^ content in the leaves and roots, and increased the K^+^, Mg^2+^ and Ca^2+^ content and antioxidant enzyme levels in the leaves and roots, thereby reducing the salt toxicity of seedlings. It showed that ABA is beneficial for protective membrane lipid peroxidation and enhances salt tolerance by regulating antioxidant defense systems and endogenous hormone balance [59]. ABA enhances tolerance to alkaline stress by initiating antioxidant defense system in roots of rice seedlings and upregulating tolerance-related genes [60]. In this study, in the comparison group of 200 mM NaCl-48 h vs. 200 mM NaCl + 10 mM KCl-48 h, the numbers of up-regulated genes for abscisic acid and auxin were 8 and 15, and the numbers of down-regulated genes were 2 and 27, respectively. In the 200 mM NaCl-168 h vs. 200 mM NaCl + 10 mM KCl-168 h comparison group, the numbers of up-regulated genes for abscisic acid and auxin were 7 and 28, and the numbers of down-regulated genes were 3 and 14, respectively. Six genes of abscisic acid showed an upward trend during 48 h and 168 h of exogenous potassium application in the roots of *T. ramosissima*, namely *Unigene0079211*, *Unigene0063711*, *Unigene0008844*, *Unigene0080236*, *Unigene0044630* and *Unigene0071368*. In particular, the expression levels of four genes, *Unigene0079211*, *Unigene0008844*, *Unigene0080236* and *Unigene0044630*, increased at 168 h, increasing the content of abscisic acid under NaCl stress. The results showed that adding exogenous potassium improved plants’ ability to absorb K^+^, and the expression levels of abscisic acid-related genes were mainly up-regulated at 48 h and 168 h under NaCl stress, which were involved in resistance to NaCl stress.

Auxin plays a key regulatory role in plant response to salt stress [61]. In wheat roots, overexpression of the auxin-related gene (*TaSAUR75*) increases salt tolerance in *Arabidopsis* [62]. In addition, Aux/IAA proteins in auxin and ARF transcription factors directly regulate auxin-responsive gene expression, and *OsIAA24* and *OsIAA20* are up-regulated in rice under high salt stress [63]. In this study, in the comparison group of 200 mM NaCl-48 h vs. 200 mM NaCl + 10 mM KCl-48 h, the number of auxin up-regulated genes was 15, and the number of down-regulated genes was 27. In the 200 mM NaCl-168 h vs. 200 mM NaCl + 10 mM KCl-168 h comparison group, the number of auxin up-regulated genes was 28, and the number of down-regulated genes was 14. Particularly, the expression levels of 4 auxin-related genes (*Unigene0000292*, *Unigene0008042*, *Unigene0048945* and *Unigene0073282*) were decreased in 200 mM NaCl-48 h vs. 200 mM NaCl + 10 mM KCl-48 h, but the expression levels of 4 genes increased in the 200 mM NaCl-168 h vs. 200 mM NaCl + 10 mM KCl-168 h comparison group. It is worthwhile to note that the expression levels of auxin-related genes (*Unigene0071678*, *Unigene0072291*, *Unigene0018027*, *Unigene0018885*, *Unigene0038393*, *Unigene0050676*, *Unigene005939*, *Unigene0070335*, *Unigene0073285* and *Unigene0077555*) were up-regulated at 48 h and 168 h of exogenous potassium application under NaCl stress. It is indicated that these differentially expressed genes may play a role in alleviating NaCl injury and are key candidate genes related to auxin in *T. ramosissima*. Especially, the Log_2_ fold-change of *Unigene0018885*, *Unigene0038393*, *Unigene0050676*, *Unigene0059399*, *Unigene0070335*, *Unigene0073285* and *Unigene0077555* increased more at 168 h. In addition, under NaCl stress, application of K and P or foliar spraying of IAA may be able to improve the salt tolerance of plants and reduce the adverse effects of salt on plants [64]. It is inferred from this information that under the influence of exogenous potassium addition, a large amount of K^+^ may be absorbed by the roots in 168 h, which increases the K^+^/Na^+^ ratio under NaCl stress and drives the up-regulation of auxin-related genes of *T. ramosissima* and promotes the growth of *T. ramosissima*.

JA is a key signaling molecule during plant development and defense [65,66], and it can play an important role in maintaining ion homeostasis [67]. Under salt stress, exogenous MeJA decreased the uptake of Na^+^ and increased the uptake of Mg^2+^, Ca^2+^ and K^+^ in rice under salt stress [68]. In this study, the expression levels of JA-related genes were up-regulated in the 200 mM NaCl-48 h vs. 200 mM NaCl + 10 mM KCl-48 h comparison group. It was shown that when *T. ramosissima* was exposed to exogenous potassium for 48 h under NaCl stress, JA decreased the absorption of Na^+^ and increased the absorption of K^+^, and actively resisted the damage of NaCl. However, due to the presence of JA in the mature leaves and roots of higher plants, the content is very low [69]. By this token, this may be the reason for the down-regulation of JA-related gene expression levels in the 200 mM NaCl-168 h vs. 200 mM NaCl + 10 mM KCl-168 h comparison group in *T. ramosissima*.

ET is an important part of plant hormones in response to salt stress, and it uses an ethylene signaling pathway to participate in plant responses to salt stress [70]. Application of ET or ACC can improve plant tolerance to high salinity [71], mainly by enhancing the expression of reactive oxygen species (ROS) scavengers [72]. *T. ramosissima* was treated with exogenous potassium for 48 h and 168 h under NaCl stress, the activity of antioxidant enzymes in leaves was increased, and the up-regulated expression of genes related to antioxidant enzyme activity enhanced its salt tolerance [53]. In cotton, *ACO* for ET biosynthesis were upregulated under both short- and long-term salt treatments and increased salt tolerance [73]. Ethylene-related gene (*Unigene0010277*) was consistently up-regulated in the 200 mM NaCl-48 h vs. 200 mM NaCl + 10 mM KCl-48 h and 200 mM NaCl-168 h vs. 200 mM NaCl + 10 mM KCl-168 h comparison groups in this study. It indicated that *Unigene0010277* played an important role in improving the salt tolerance of *T. ramosissima*.

Cytokinin (CK) is a major regulator of plant growth and development that controls plant adaptation to salt stress [74,75,76]. In particular, some plants’ up-regulated expression of cytokinin-related genes mitigates the damage caused by salt stress [77,78]. *Tamarix hispida* salt tolerance was reduced by RNAi expression of *ThCRF1* under salt stress. Despite this, *ThCRF1* overexpression significantly enhanced salt tolerance of *Tamarix hispida* by controlling osmotic potential and activating antioxidant enzymes [79]. In this study, the expression level of 1 CK gene (*Unigene0045738*) in the 200 mM NaCl-48 h vs. 200 mM NaCl + 10 mM KCl-48 h and 200 mM NaCl-168 h vs. 200 mM NaCl + 10 mM KCl-168 h comparison groups was up-regulated, and *Unigene0045738* increased more at 168 h Log_2_ fold-change. Results showed that cytokinin actively resists salt stress when roots of *T. ramosissima* are exposed to exogenous potassium for 48 h and 168 h under NaCl stress. Notably, Unigene0045738 is a key candidate gene for cytokinin in response to NaCl stress.

## 5. Conclusions

During 48 h and 168 h of exogenous potassium application in *T. ramosissima* roots under NaCl stress, the synthesis, signal transmission and metabolism of various plant hormones participated in the construction of a defense system, and a large number of plant hormones regulate their related genes to actively participate in resistance to NaCl, maintain plant nutritional homeostasis, and ensure normal plant growth. In particular, the interaction between different plant hormones maintains ionic homeostasis by reducing Na^+^ accumulation, increasing K^+^ uptake, maintaining ionic homeostasis, and better maintaining K^+^/Na^+^ ratio balance, enhancing the activity of antioxidant enzymes, thereby alleviating the damage caused by NaCl. It is worth noting that the plant hormone signal transduction pathway plays an important role in alleviating NaCl stress, but the mechanism of its related gene regulation and the metabolites that alleviate NaCl toxicity remain to be further explored.

To sum up, the important metabolic pathways and key candidate genes related to plant hormones in response to NaCl stress of *T. ramosissima* after 48 h and 168 h of exogenous potassium application can provide a scientific theoretical basis for the breeding of salt-tolerant varieties and the mitigation of NaCl toxicity by K^+^.

## Figures and Tables

**Table 1:** Top 10 KEGG pathways.

Number	Pathway	Pathway Annotated Genes	*p*-Value	Pathway ID
200 mM NaCl-48 h vs. 200 mM NaCl + 10 mM KCl-48 h				
1	Photosynthesis	83	0.000000	ko00195
2	Ribosome	703	0.000000	ko03010
3	Flavonoid biosynthesis	25	0.000000	ko00941
4	Photosynthesis—antenna proteins	52	0.000000	ko00196
5	Phenylpropanoid biosynthesis	63	0.000000	ko00940
6	Plant hormone signal transduction	74	0.000079	ko04075
7	Stilbenoid, diarylheptanoid and gingerol biosynthesis	12	0.000824	ko00945
8	Zeatin biosynthesis	9	0.004980	ko00908
9	Cutin, suberine and wax biosynthesis	11	0.008045	ko00073
10	Carotenoid biosynthesis	15	0.011468	ko00906
200 mM NaCl-168 h vs. 200 mM NaCl + 10 mM KCl-168 h				
1	Ribosome	703	0.000000	ko03010
2	Phenylpropanoid biosynthesis	57	0.000000	ko00940
3	Plant hormone signal transduction	70	0.000002	ko04075
4	Zeatin biosynthesis	11	0.000042	ko00908
5	Glycerolipid metabolism	44	0.001344	ko00561
6	Brassinosteroid biosynthesis	6	0.005758	ko00905
7	Flavonoid biosynthesis	12	0.007853	ko00941
8	Cutin, suberine and wax biosynthesis	9	0.020172	ko00073
9	Monoterpenoid biosynthesis	2	0.020236	ko00902
10	Diterpenoid biosynthesis	7	0.021957	ko00904

**Table 2:** Plant hormone candidate gene for exogenous potassium application in *T. ramosissima* roots under NaCl stress.

Pathway	Gene ID	Description	Log_2_ Fold-Change	
			200 mM NaCl-48 h vs. 200 mM NaCl + 10 mM KCl-48 h	200 mM NaCl-168 h vs. 200 mM NaCl + 10 mM KCl-168 h
Abscisic Acid				
ko00906	*Unigene0079211*	Abscisic acid 8′-hydroxylase 2-like	0.44	4.17
	*Unigene0063711*	Abscisic acid 8′-hydroxylase	4.62	1.50
	*Unigene0008844*	Abscisic acid 8′-hydroxylase 2-like	0.58	2.74
	*Unigene0027741*	Abscisic acid 8′-hydroxylase CYP707A2 isoform X1	1.70	−0.01
ko04016; ko04075	*Unigene0080236*	Abscisic acid receptor PYL2-like	0.05	7.85
	*Unigene0039063*	Abscisic acid receptor PYR1-like	−0.49	0.28
	*Unigene0044630*	Abscisic acid receptor PYL4-like	0.44	1.14
	*Unigene0054686*	Abscisic acid receptor PYR1-like	2.01	−0.97
	*Unigene0071368*	Abscisic acid receptor PYL4	2.09	0.03
ko04075	*Unigene0028134*	Abscisic acid-insensitive 5-like protein 2	−1.79	−1.33
Auxin				
ko04075	*Unigene0000292*	Auxin-responsive protein IAA14-like	−0.25	0.06
	*Unigene0003420*	Auxin response factor 3-like	−1.73	−1.08
	*Unigene0008042*	Auxin-responsive protein IAA14	−1.51	0.50
	*Unigene0009423*	Auxin	−0.76	0.17
	*Unigene0011116*	Auxin-responsive protein SAUR32-like	−0.13	−0.07
	*Unigene0018027*	Auxin-responsive protein SAUR21	10.01	8.49
	*Unigene0018598*	Auxin-induced protein AUX22-like	−1.04	−2.11
	*Unigene0018885*	Auxin-responsive protein SAUR36	1.16	2.07
	*Unigene0019018*	Auxin-responsive protein SAUR50-like	−3.55	−0.04
	*Unigene0029265*	Auxin-induced protein 22D-like	−0.35	2.06
	*Unigene0030611*	Auxin-responsive protein SAUR50	7.32	−9.42
	*Unigene0031005*	Auxin response factor 1-like	−0.98	−0.12
	*Unigene0038393*	Auxin-responsive protein SAUR72	0.34	0.69
	*Unigene0046011*	Auxin-responsive protein SAUR32	−0.01	1.59
	*Unigene0047953*	Auxin-responsive protein SAUR32	−0.78	0.27
	*Unigene0048320*	Auxin response factor 9-like	−0.07	0.37
	*Unigene0048945*	Auxin-induced protein 15A-like	−2.51	0.37
	*Unigene0050061*	Auxin-responsive protein IAA9	−1.17	−0.97
	*Unigene0050675*	Auxin transporter-like protein 3	−0.20	3.19
	*Unigene0050676*	Auxin transporter-like protein 3	0.23	3.33
	*Unigene0050677*	Auxin transporter-like protein 5 isoform X1	−2.08	0.11
	*Unigene0052348*	Auxin-responsive protein IAA29-like	1.39	−1.38
	*Unigene0054810*	Auxin-responsive protein SAUR50	0.83	−1.01
	*Unigene0059004*	Auxin-induced protein 22B	−0.13	0.23
	*Unigene0059322*	Auxin-responsive protein SAUR50	−0.08	0.70
	*Unigene0059399*	Auxin-induced protein 6B-like	0.41	0.59
	*Unigene0065338*	Auxin transporter-like protein 4	0.14	−0.09
	*Unigene0065339*	Auxin transporter-like protein 2	−0.92	0.26
	*Unigene0070335*	Auxin-responsive protein SAUR71-like	0.71	1.27
	*Unigene0071678*	Auxin-responsive protein IAA26	0.29	0.78
	*Unigene0072291*	Auxin-responsive protein IAA26-like	0.40	0.40
	*Unigene0073282*	Auxin-responsive protein IAA27-like protein	−3.44	1.20
	*Unigene0073285*	Auxin-responsive protein	0.51	9.06
	*Unigene0074040*	Auxin-responsive protein IAA31	−0.43	1.44
	*Unigene0077555*	Auxin-induced protein 6B-like	0.14	0.74
	*Unigene0085809*	Auxin-responsive protein SAUR71-like	0.98	−0.61
	*Unigene0088743*	Auxin response factor 3-like	−0.22	−0.51
	*Unigene0089917*	Auxin-responsive protein IAA8-like isoform X2	−0.23	0.55
	*Unigene0091437*	Auxin-responsive protein IAA7	−0.30	1.68
	*Unigene0097673*	Auxin response factor 11	−0.42	1.35
	*Unigene0102316*	Auxin-induced protein 15A-like	−2.88	−1.47
	*Unigene0105390*	Auxin-responsive protein SAUR71-like	−2.70	−1.07
Ethylene				
ko04016; ko04075;	*Unigene0010277*	Ethylene response factor 11	1.94	0.72
ko04626	*Unigene0080149*	Ethylene response factor 6	−0.47	−0.47
Jasmonic acid				
ko04075	*Unigene0038537*	Jasmonic acid-amido synthetase JAR1	1.82	−1.54
Cytokinin				
ko00908	*Unigene0045738*	Cytokinin oxidase/dehydrogenase	1.87	3.55

**Table 3:** Information sheet for 25 species.

Family	Species	Description	Protein ID	CDS (bp)	ORF Length (aa)
Euphorbiaceae	*Ricinus communis*	Auxin-responsive protein IAA14	XP_002517023.3	711	236
Actinidiaceae	*Actinidia chinensis* var. *chinensis*	Auxin-responsive protein	PSS33813.1	681	226
Anacardiaceae	*Pistacia vera*	Auxin response factor 5	XP_031270171.1	2841	946
Amaranthaceae	*Chenopodium quinoa*	Auxin-induced protein 22D-like	XP_021742258.1	546	181
Cucurbitaceae	*Cucumis sativus*	Auxin-responsive protein SAUR32	XP_011656853.1	303	100
Solanaceae	*Capsicum chinense*	Auxin-responsive protein SAUR32	PHT98376.1	501	166
Pedaliaceae	*Sesamum indicum*	Auxin response factor 9	XP_011074533.1	1890	629
Rosaceae	*Pyrus bretschneideri*	Auxin-responsive protein SAUR50	XP_009335221.1	318	105
Oleaceae	*Olea europaea* subsp. *Europaea*	Auxin transporter 3	CAA3012958.1	1398	465
Fagaceae	*Quercus suber*	Auxin transporter-like protein 5	XP_023883666.1	1473	490
Anacardiaceae	*Mangifera indica*	Auxin-induced protein 22B-like	XP_044499499.1	570	189
Vitaceae	*Vitis vinifera*	Auxin-responsive protein SAUR50	RVW80229.1	561	186
Euphorbiaceae	*Manihot esculenta*	Auxin transporter-like protein 2	XP_021623668.1	1452	483
Proteaceae	*Telopea speciosissima*	Auxin-responsive protein IAA27	XP_043724596.1	981	326
Rosaceae	*Rosa chinensis*	Auxin-responsive protein IAA31	XP_024192080.1	633	210
Rosaceae	*Malus domestica*	Auxin responsive protein	AZI15332.1	1092	363
Brassicaceae	*Brassica napus*	Auxin-responsive protein IAA7	XP_013749219.1	729	242
Amaranthaceae	*Spinacia oleracea*	Auxin response factor 11	XP_021846673.1	1995	664
Papaveraceae	*Macleaya cordata*	Auxin-induced protein	OVA12640.1	285	94
Malvaceae	*Durio zibethinus*	Auxin-induced protein 6B-like	XP_022772137.1	438	145
Theaceae	*Camellia sinensis*	Auxin-responsive protein SAUR71-like	XP_028108662.1	402	133
Myricaceae	*Morella rubra*	Auxin-responsive protein IAA26	KAB1218495.1	1098	365
Rosaceae	*Prunus avium*	Auxin-responsive protein IAA26-like	XP_021818768.1	1080	359
Malvaceae	*Herrania umbratica*	Auxin-responsive protein IAA27	XP_021276374.1	919	312
Cucurbitaceae	*Momordica charantia*	Auxin-induced protein 6B-like	XP_022138000.1	453	150

## Data Availability

Not applicable.

## Supplementary Materials

The following supporting information can be downloaded at: https://www.mdpi.com/article/10.3390/genes13101803/s1. Supplementary Table S1: Sequences of specific primers. Supplementary Table S2: Annotation of key candidate genes of plant hormone signal transduction pathway. Supplementary Figure S1: Statistics of differentially expressed genes. Supplementary Figure S2: Expression levels of genes co-annotated in plant hormone signal transduction pathway at 48 h and 168 h. Supplementary Figure S3: Phylogenetic tree analysis of *T*. *ramosissima* auxin and other species auxins.
