# Supplementary material for: Effects of Exogenous (K+) Potassium Application on Plant Hormones in the Roots of Tamarix ramosissima under NaCl Stress
Source: Genes (Basel). 2022 Oct 6;13(10):1803. doi: 10.3390/genes13101803 (PMC9601537; doi:10.3390/genes13101803)
Supplement: Supplementary file 1 [file genes-13-01803-s001.zip › Supplementary Figure S2.pdf]

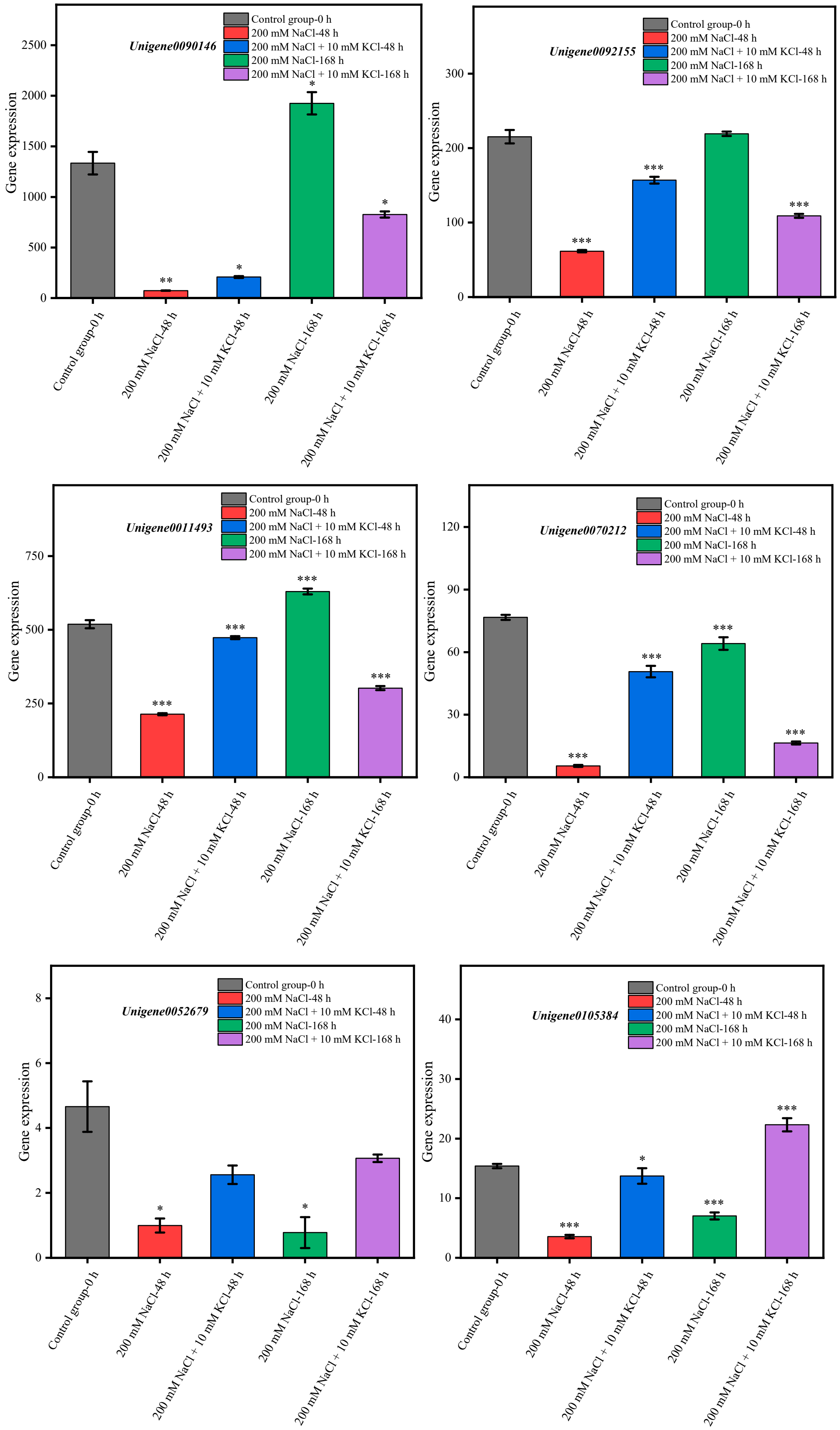
Unigene0090146
Control group-0 h
200 mM NaCl-48 h
200 mM NaCl + 10 mM KCl-48 h
200 mM NaCl-168 h
200 mM NaCl + 10 mM KCl-168 h
Gene expression
0
500
1000
1500
2000
2500
**
*
*
*
Control group-0 h
200 mM NaCl-48 h
200 mM NaCl + 10 mM KCl-48 h
200 mM NaCl-168 h
200 mM NaCl + 10 mM KCl-168 h
Unigene0092155
Gene expression
0
100
200
300
***
***
***
Unigene0011493
Gene expression
0
250
500
750
***
***
***
***
Unigene0070212
Gene expression
0
30
60
90
120
***
***
***
***
Unigene0052679
Gene expression
0
2
4
6
8
*
*
Unigene0105384
Gene expression
0
10
20
30
40
***
*
***
***

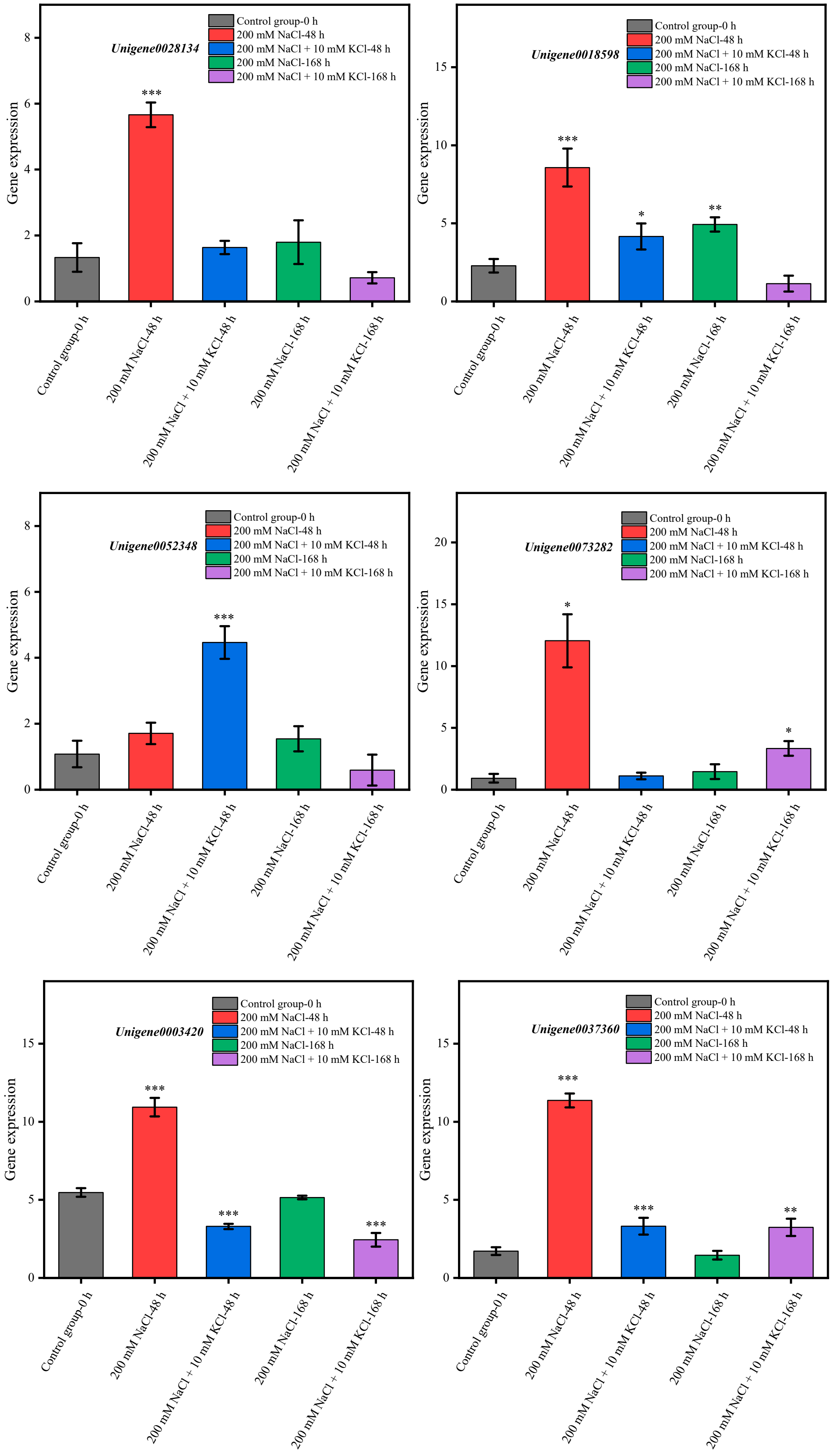
Unigene0028134
Unigene0018598
Unigene0052348
Unigene0073282
Unigene0003420
Unigene0037360
Gene expression
Control group-0 h
200 mM NaCl-48 h
200 mM NaCl + 10 mM KCl-48 h
200 mM NaCl-168 h
200 mM NaCl + 10 mM KCl-168 h

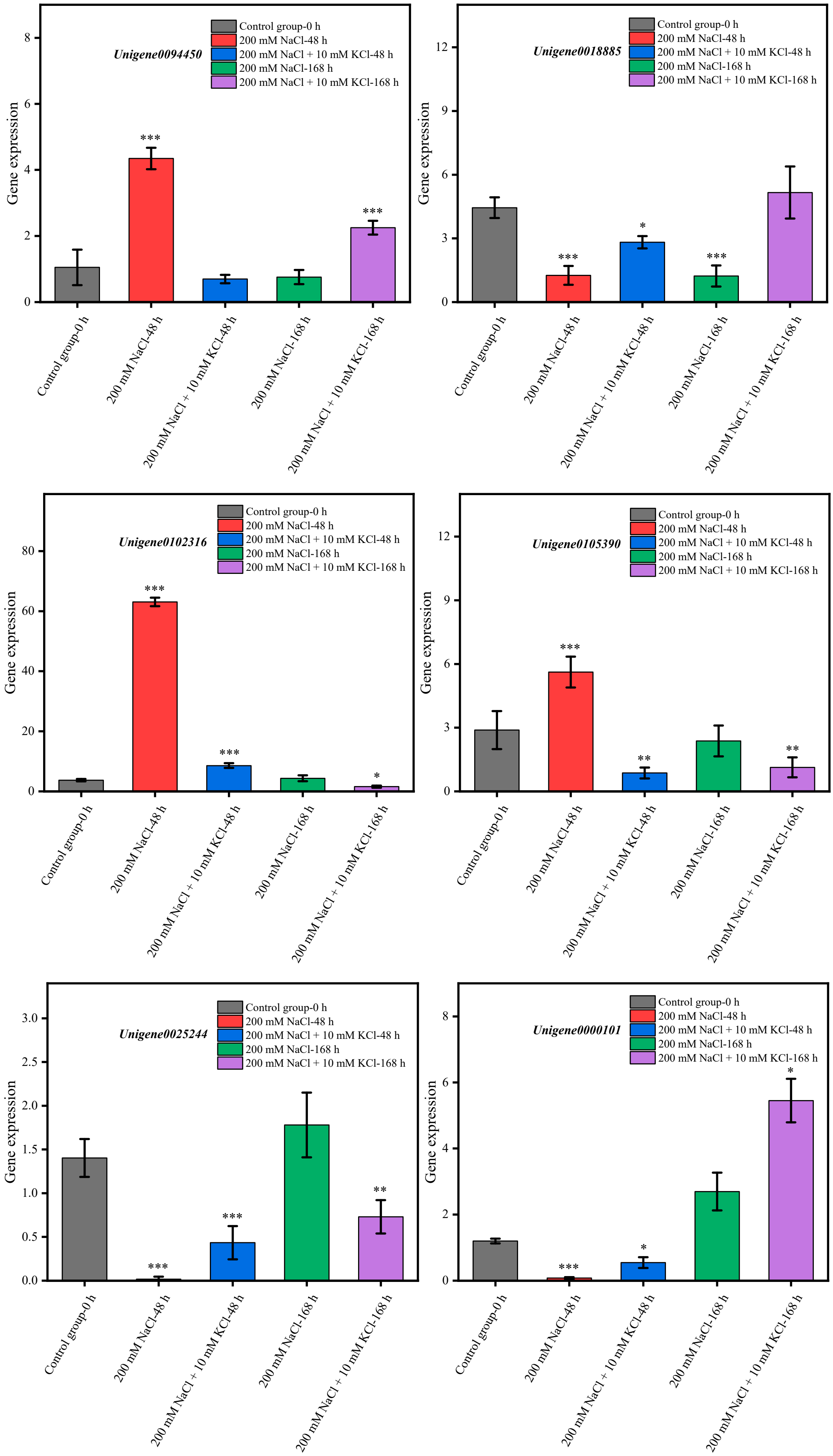
Unigene0094450
Unigene0018885
Unigene0102316
Unigene0105390
Unigene0025244
Unigene0000101
Control group-0 h
200 mM NaCl-48 h
200 mM NaCl + 10 mM KCl-48 h
200 mM NaCl-168 h
200 mM NaCl + 10 mM KCl-168 h
Gene expression
***
**
*

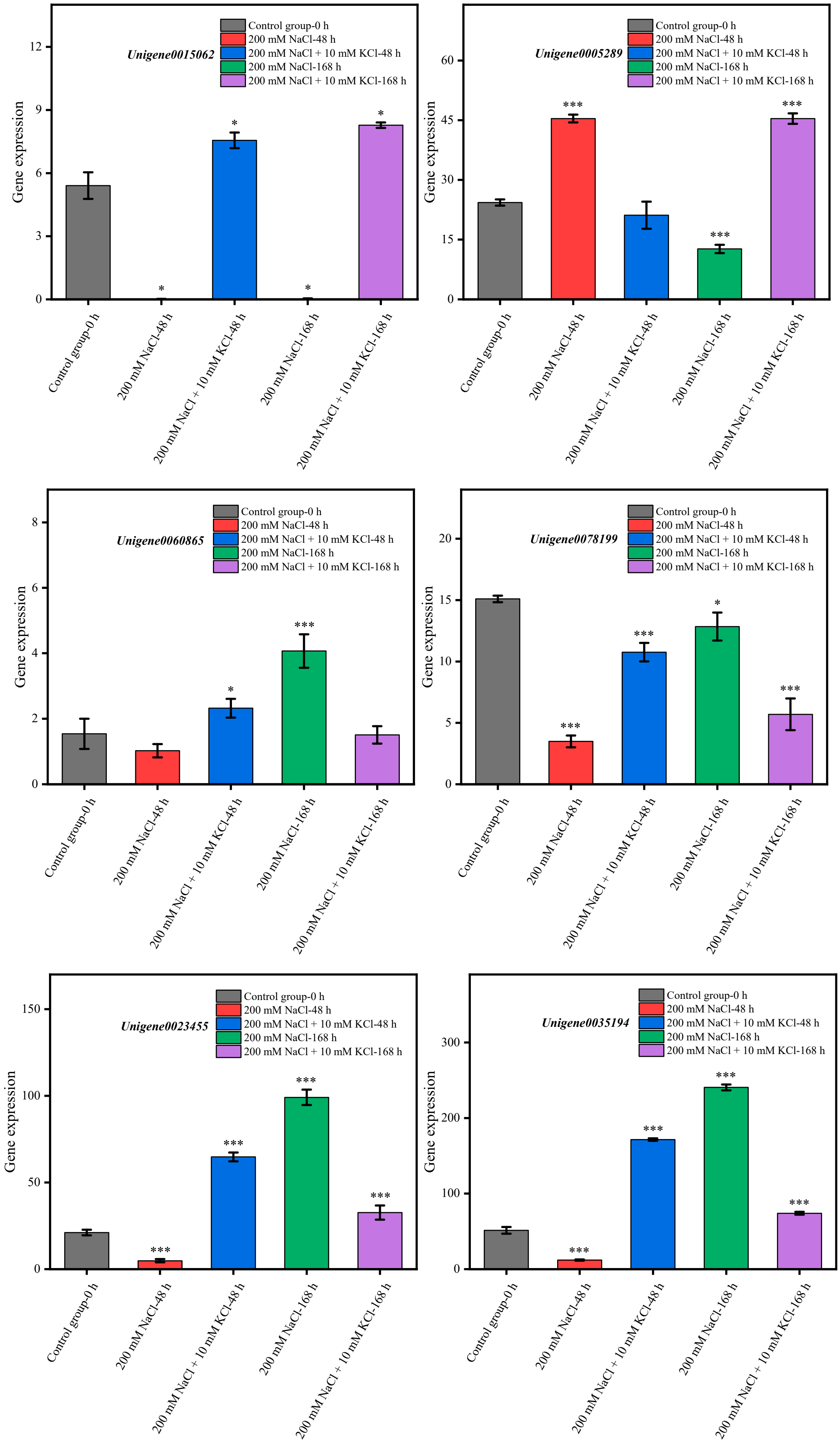
Unigene0015062
Unigene0005289
Unigene0060865
Unigene0078199
Unigene0023455
Unigene0035194
Control group-0 h
200 mM NaCl-48 h
200 mM NaCl + 10 mM KCl-48 h
200 mM NaCl-168 h
200 mM NaCl + 10 mM KCl-168 h
Gene expression
*
***

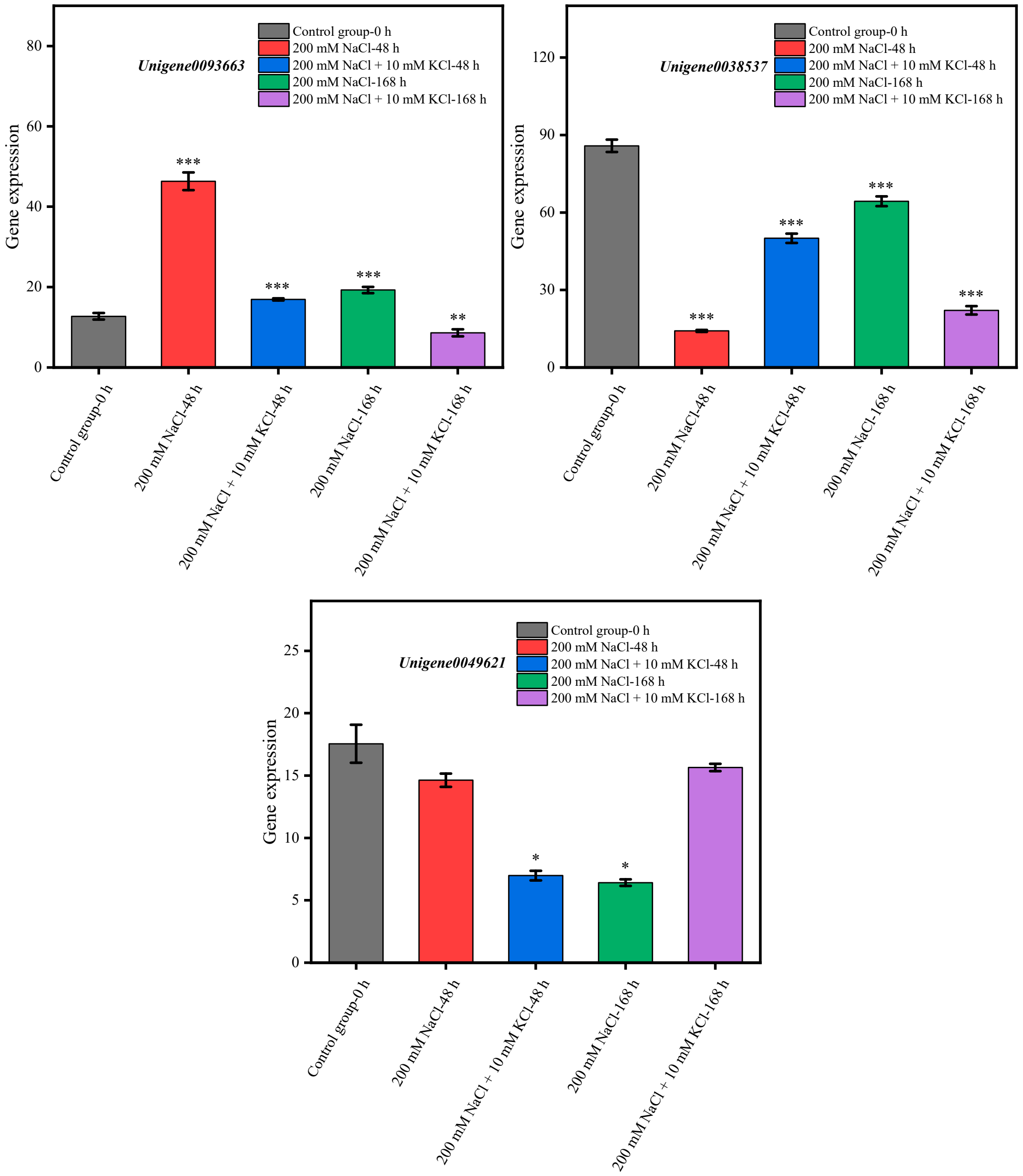

Supplementary Figure S2. Expression levels of genes co-annotated in plant hormone signal transduction pathway at 48 h and 168 h

(In the plant hormone signal transduction pathway, 27 genes were co-annotated to the 200 mM NaCl-48h vs. 200 mM NaCl + 10 mM KCl-48h and 200 mM NaCl-168h vs. 200 mM NaCl + 10 mM KCl-168h comparison groups, and they had different expression levels).
