# Supplementary material for: Effects of Exogenous (K+) Potassium Application on Plant Hormones in the Roots of Tamarix ramosissima under NaCl Stress
Source: Genes (Basel). 2022 Oct 6;13(10):1803. doi: 10.3390/genes13101803 (PMC9601537; doi:10.3390/genes13101803)
Supplement: Supplementary file 1 [file genes-13-01803-s001.zip › Supplementary Figure S3.pdf]

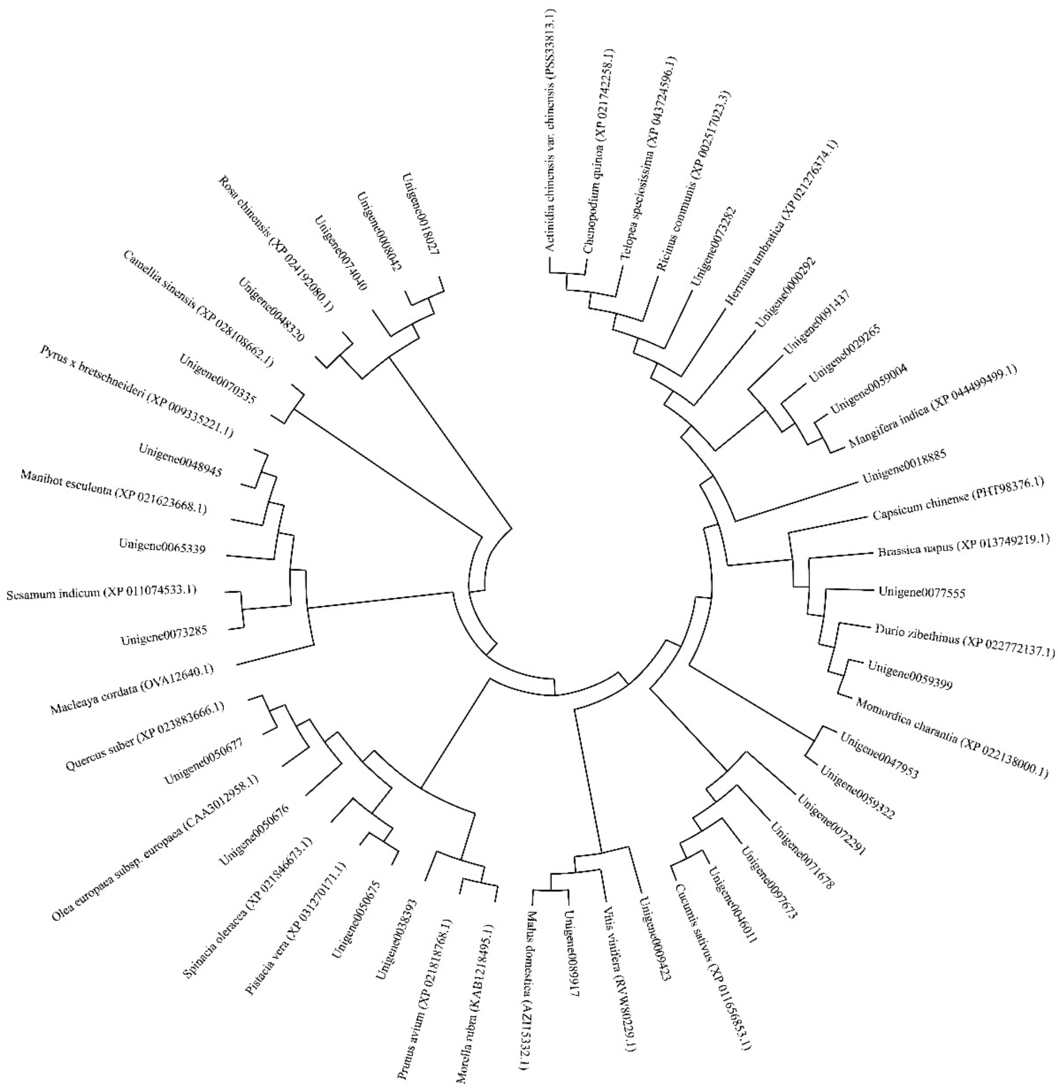

Supplementary Figure S3. Phylogenetic tree analysis of *T. ramosissima* auxin and other species auxins (A phylogenetic tree was constructed for analysis of the protein amino acid sequences of 28 auxins from *T. ramosissima* root and the protein amino acid sequences of other 25 species).
