# Supplementary material for: Effects of Exogenous (K+) Potassium Application on Plant Hormones in the Roots of Tamarix ramosissima under NaCl Stress
Source: Genes (Basel). 2022 Oct 6;13(10):1803. doi: 10.3390/genes13101803 (PMC9601537; doi:10.3390/genes13101803)
Supplement: Supplementary file 1 [file genes-13-01803-s001.zip › Supplementary Table S1.pdf]

Supplementary Table S1. Sequences of specific primers

Primer name	Primer sequence (5'-3')
Unigene0073282	F: TGGAGCATGATTACATAGGATTATCAGAGG
	R: AGTCTTAACTCGGTGGCCTTGAAG
Unigene0037360	F: GGTCAATCTCAATCCGCTGTGTAAG
	R: TGTTCCTTCTCAGTAAGTGCCTTGG
Unigene0005289	F: TTAAGATTGGCAGCGTAAGGGAAGTAA
	R: AAGATATGCTCCTCGTCGTCAAGAAG
Unigene0049621	F: CACTGACTGAATCTCTTCCAGACGAAT
	R: TCATCCTTGCGAATGCTACTCAATAGA
Unigene0018885	F: AGAACGAAGATGGCGGCTGGTAT
	R: CCGAATTTCTCCTCTGCTTCTCTCAAC
Unigene0010277	F: ACACTCCTCGTCGGAATCCTCATC
	R: TCGTTCAGCATGTCGAATAGAACCATT
Unigene0045738	F: GTATTCAGCCGACGACGTAGCC
	R: CATCTCGACCACCACGCCATTAC
Tubulin	F: GCTGAGATTACAACCGCTG
	R: CTGTTCGTTTGGTCTTGATT

Note: F means Forwardprimer, R means Reversedprimer.
