# Supplementary material for: Effects of Exogenous (K+) Potassium Application on Plant Hormones in the Roots of Tamarix ramosissima under NaCl Stress
Source: Genes (Basel). 2022 Oct 6;13(10):1803. doi: 10.3390/genes13101803 (PMC9601537; doi:10.3390/genes13101803)
Supplement: Supplementary file 1 [file genes-13-01803-s001.zip › Supplementary Table S2.pdf]

Supplementary Table S2. Annotation of key candidate genes of plant hormone signal transduction pathway

Gene ID	Description	Pathway
Unigene0052679	transcription factor TGA9-like isoform X3	ko04075
Unigene0105384	transcription factor HBP-1b(c38)-like	ko04075
Unigene0073282	auxin-responsive protein IAA27-like protein	ko04075
Unigene0037360	GH3 auxin-responsive promoter	ko04075
Unigene0094450	PREDICTED: jasmonic acid-amido synthetase JAR1-like	ko04075
Unigene0018885	Auxin-responsive protein SAUR36	ko04075
Unigene0000101	Histidine-containing phosphotransfer protein	ko04075
Unigene0015062	PREDICTED: two-component response regulator ARR2-like isoform X3	ko04075
Unigene0005289	regulatory component of ABA receptor3-like	ko04016; ko04075
Unigene0049621	PREDICTED: EIN3-binding F-box protein 1-like	ko04016; ko04075
